# Effect of Vibration Direction on Two-Dimensional Ultrasonic Assisted Grinding-Electrolysis-Discharge Generating Machining Mechanism of SiCp/Al

**DOI:** 10.3390/ma16072703

**Published:** 2023-03-28

**Authors:** Jing Li, Wanwan Chen, Yongwei Zhu

**Affiliations:** 1 School of Mechanical Engineering, Yangzhou University, Yangzhou 225127, China; 2 JITRI Institute of Precision Manufacturing, Nanjing 211800, China

**Keywords:** vibration direction, grinding electrolysis discharge, grinding force, surface roughness

## Abstract

This study proposes the mechanism of two-dimensional ultrasonic assisted grinding- electrolysis-discharge generating machining (2UG-E-DM). It analyzed the influence of vibration directions on grinding characteristics and surface morphology through the motion simulation of an abrasive. Comparative experiments with different vibration directions verified the effect of ultrasonic assistance on the weakening of the grinding force, the widening of the surface pits, and the leveling of the surface morphology of SiC_p_/Al composites. Simulation analysis of a single abrasive particle verified the test results. The results of machining tests at different amplitudes showed that as the workpiece and tool amplitude increased, the grinding force of the normal force decreased faster than that of the tangential force. The effect of surface electrolysis discharge machining was significant, and the number of exposed particles increased, but the residual height of the surface and the surface roughness were reduced by vibration grinding. When the two-dimensional amplitude was increased to 5 μm, the axial and tangential vibrations increased the grinding domain, and the dragging and rolling of the reinforced particles significantly reduced the surface roughness, which obtained good surface quality.

## 1. Introduction

Aluminum-based silicon carbide composite materials are increasingly used in the field of turbine part material preparation due to their various advantages, such as high hardness, low weight, and wear resistance [1,2]. Traditional grinding, milling, and other mechanical processing technologies cannot be processed efficiently and with high quality; however, ultrasonic-assisted processing technology, which is similar to “micro-grinding”, performs remarkably well on hard, brittle, and difficult-to-machine materials and can generate better workpiece surface quality while utilizing lower grinding force [3,4,5,6]. Electrolytic machining (ECM) is suitable for the processing of curved surfaces and tiny holes of conductive materials because of its non-contact force and high processing efficiency, but electrical discharge machining (EDM) is often selected for the processing of non-conductive materials [7,8,9]. Therefore, it is necessary to take full use of the advantages of various processing technologies, change the process method used, and utilize ultrasonic assistance in different vibration directions to improve the processing effect of composite materials [10,11,12].

The direction of ultrasonic vibration affects the grinding force and surface topography [13]. Many studies have added ultrasonic vibration to tools to improve grinding performance when faced with difficult-to-machine composite materials [14]. Shi [15] established a CFRP composite material ultrasonic side grinding force prediction model based on the brittle–ductile material removal mechanism. The grinding force of ultrasonic-assisted grinding was smaller than general grinding, while the frequency and amplitude of ultrasonic vibrations were related to the grinding force, and a close relationship was shown between them. The grinding force prediction model was consistent with the variation trend of the experimental values. Cong [16] regarded CFRP composites as equivalent homogeneous materials and established a prediction model for the brittleness removal of grinding force using rotating ultrasonic grinding based on the depth of abrasive indentation. The influence of effective abrasive particles and the impact of ultrasonic vibration on grinding were taken into account. However, only the effects of axial ultrasonic amplitude on indentation depth, effective cutting times, and the maximum impact force were studied. Li [17] established a grinding force model for rotating the ultrasonic surface machining of C/SiC composites based on dynamic analysis and proposed the conditions for intermittent processing and found that the grinding force of intermittent processing was 30% lower than general grinding. However, the relationship between vibration direction and surface quality after grinding was not considered.

Zha [18] analyzed ultrasonic vibrations according to the scribing morphology and scribing force and they increased the fracture cracks of SiC particles and reduced the friction coefficient. The surface of the metal-based materials was continuous and smooth in ultrasonic-assisted grinding while the reinforcement particles were partially broken and partially peeled off to form pits. Wang [19] analyzed the surface morphology generation mechanism of tangential ultrasonic-vibration-assisted grinding. The abrasive repeatedly rolled the machined surface to make the machined surface smoother. Gao [20] analyzed the “broadening effect” of axial ultrasound in two-dimensional ultrasonic amplitude-assisted grinding using multi-angle two-dimensional surface characterization tests and found that it reduced or eliminated the protrusion height of adjacent grooves. Additionally, Liu [21] studied groove widening by axial vibration and rolling by tangential ultrasonic vibration and found that two-dimensional ultrasonic vibration had more advantages than one-dimensional vibration. However, currently, there is no comparative study of different vibration directions. Under the joint actions of electric spark erosion, electrochemical corrosion, and mechanical grinding, surface roughness (Ra) was an order of magnitude that was lower than that of a single process [22]. The above studies did not consider the influence of vibration directions and their errors regarding machining performance. When the vibration direction is inconsistent with the machining direction, ultrasonic vibration cannot play a role in reducing the grinding force, and the grinding will not reduce the surface roughness; rather, it will cause damage or destruction to the machining surface.

2UG-E-DM has the advantages of ultrasonic hammering in reducing grinding force, rolling, and leveling a machined surface. Therefore, it is necessary to further study the effect of vibration direction on machining effect. This paper innovatively proposes the material removal mechanism of abrasive particles on a 2UG-E-DM machining surface using different vibration directions and verifies the advantages of significantly reducing the grinding force and surface roughness under the assistance of two-dimensional ultrasonic vibration directions through comparison tests, different parameter tests, and simulation analysis.

## 2. Machining Mechanism of 2UG-E-DM

**Figure 1:**
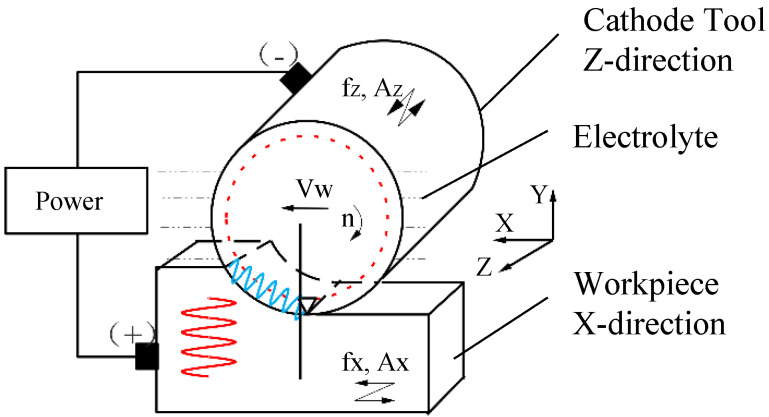
The machining mechanism of 2UG-E-DM.

The processing principle of 2UG-E-DM, which is based on ultrasonic vibration technology and electric processing technology, is shown in Figure 1. The XYZ coordinate system is established based on the position of the center of the end face during tool machining. The initial vibration direction of the workpiece is the X direction (or tangential direction), and the vibration amplitude and frequency are $A_{X}$ and $f_{X}$, respectively. The X direction and Y direction translational feeds are followed with the system working platform. The spindle speed is n; the feed rate is v_w_; the ultrasonic vibration (or axial direction) is along the Z axis; the amplitude and frequency are $A_{Z}$ and $f_{Z}$, respectively; the grinding depth is $a_{p}$; the initial cutting line of the tool abrasive ends with the workpiece surface; and the cutting line leaves the grinding processing area. The workpiece material is connected to the positive pole of the power supply; the cathode tool is coated with diamond abrasive and connected to the negative pole of the power supply; and the processing area is immersed in a passive electrolyte. The motion equation of an abrasive on a tool and a workpiece or $S_{P}\left(t\right)$ and $S_{W}\left(t\right)$, respectively, can separately be expressed by Equations (1) and (2).

(1) $$S_{P}\left(t\right)=\{\begin{array}{c} v_{W}t+\mathrm{Rsin}\left(\frac{\pi n}{30}t\right)\\ -R\cos\left(\frac{\pi n}{30}t\right)\\ A_{Z}\sin\left(2{\pi f}_{Z}t\right) \end{array}$$

(2) $$S_{W}\left(t\right)=A_{X}\sin\left(2{\pi f}_{X}t\right)$$

**Figure 2:**
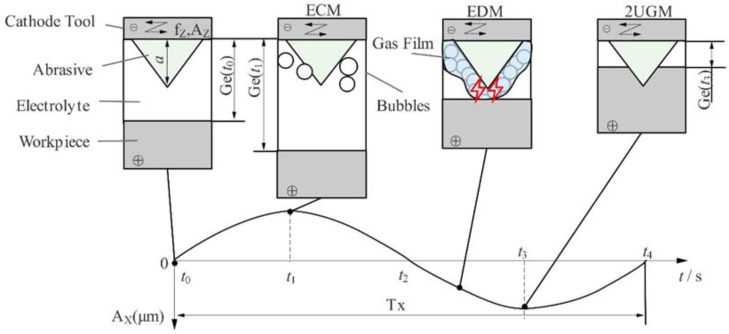
The machining process within a vibration cycle of the workpiece.

2UG-E-DM is an improved method based on two-dimensional ultrasonic assisted grinding (2UGM), electrolysis machining (ECM), and electrical discharge machining (EDM). The workpiece and cathode tool periodically change the vibration displacement; therefore, the three processing processes of two-dimensional ultrasonic-assisted electrolysis, discharge, and grinding do not always occur at the same time rather than intermittent processing under certain conditions. In the time of $t_{0}\to t_{4}$, the machining process inside a vibration period ($T_{X}$) of the workpiece is shown in Figure 2. When the two poles are powered on, the gap $G_{e}\left(t\right)$ between the electrolytic electrodes decreases in the workpiece vibration time $t_{1}\to t_{3}$, while the current density and the electrolytic speed of the metal base increases achieve a higher material removal rate at a low voltage and show more localization processing capacity. During the time periods of $t_{0}\to t_{1}$ and $t_{3}\to t_{4}$, the gap $G_{e}\left(t\right)$ becomes larger, the current density decreases, the electrolysis speed of the metal-based materials decreases, and the pressure between the electrodes decreases rapidly. Under the effects of pumping, cavitation, and eddy current, the electrolyte is renewed and circulated, and solid, processed products are flushed out, which improves and stabilizes the processing environment [23]. There is always a minimum electrolytic gap between the cathode tool substrate and the workpiece (which is approximately equal to the height a of an abrasive and much larger than the diameter of the SiC particle), and there is no direct contact between the working electrodes; in turn, this avoids the occurrence of an electrolytic “short circuit”. Under the actions of cathode tool rotation and ultrasonic vibration, the bubbles generated by electrolysis gradually gather and cover the cathode tool to form a gas film, and a high-intensity electric field is established due to the high resistance between the electrodes. When the critical gap value is reached, the electric field breaks down the dielectric electrode of the electrolyte. When the gas film insulates the cathode tool from the surface of the workpiece, the inter-electrode gas film is similar to the insulating working medium, which generates a spark discharge. Electrolytic discharge then occurs when the gas film insulates the cathode tool from the electrolyte. The abrasive of the cathode tool touches the workpiece surface and starts to grind intermittently only when the gap $G_{e}\left(t\right)$ is less than the height of the abrasive in the time $t_{2}\to t_{4}$, which removes the material until the largest grinding force is produced in the time $t_{3}$. The workpiece material is removed in various forms, such as electrolytic dissolution, grinding, and discharge erosion. The final surface of the workpiece is tangentially ground by tools and machined by a secondary electrolysis-discharge process to become more level.

## 3. Experimental Setup

**Figure 3:**
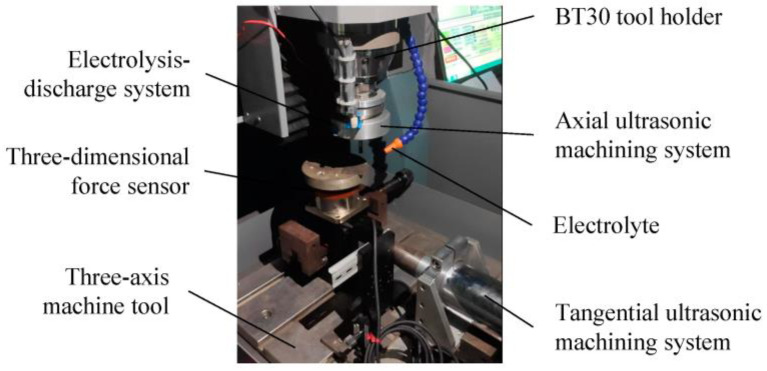
The 2UG-E-DM experimental device.

**Figure 4:**
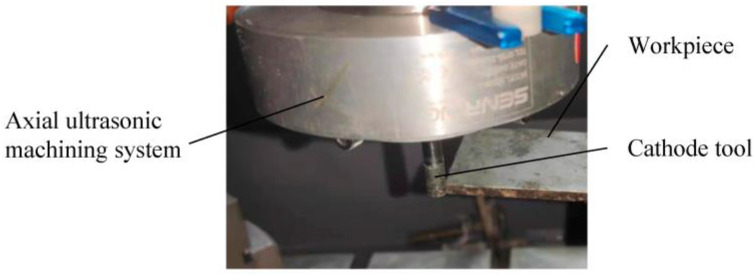
The machining area.

The 2UG-E-DM device is shown in Figure 3 which consisted of a three-axis machining machine, tangential and axial ultrasonic machining systems (the resonant frequencies were 18.33 and 19.46 kHz, respectively), and an electrolysis-discharge system. The vibration was transmitted to the workpiece through the X direction ultrasonic generator, and the Z direction ultrasonic vibration device was added to the BT30 tool holder. The cathode tool was connected to the negative pole of the power supply through a conductive slip ring, and the workpiece was connected to the positive pole of the power supply and was immersed in an electrolyte with low voltage and low current density to realize electrolysis-discharge machining [24]. The cathode tool used in this experiment was made of tungsten steel, and its diameter was 6 mm, while the surface was coated with diamond abrasives of about 150 μm. The workpiece material was silicon carbide particle reinforced aluminum matrix composite material (40% SiCp/Al), and the physical property parameters of the material are shown in Table 1. The average particle size of the silicon carbide particles was 5 μm. The initial size of the workpiece sample was 50 mm × 50 mm × 5 mm, and the processing surface of 50 mm × 5 mm was ground and cleaned before machining. The machining area is shown in Figure 4.

The electrolyte was a solution of 5 wt.% NaCO_3_ which was pumped through a nozzle during machining. A comparative test of different processes was designed, including GM (General Machining), G-E-DM (Grinding and Electrolysis Discharge Machining), XUG-E-DM (X Direction Grinding, Electrolysis, and Discharge Machining), ZUG-E-DM (Z Direction Grinding, Electrolysis, and Discharge Machining), and 2UG-E-DM. The spindle speed was 1000 r/min, the feed rate was set at 30 mm/min, and the processing depth was 0.01 mm. Other parameters are shown in Table 2. According to the comparative test results, when the voltage was 4 V, the processing tests of different amplitudes under different processing depths (0.01 and 0.02 mm) were designed as shown in Table 3.

The test was carried out three times, and the average value was taken as the test result. The three-dimensional force sensor (DZ-310) had a range of 100 N and a measurement accuracy of 0.05 N and was used to measure the tangential and axial grinding forces. A surface profiler (Counter GT-X) was used to measure the surface roughness (Ra), while an LSM 700 laser microscope was used to construct 3D topography images, and a scanning electron microscope (SEM) was used to characterize the processed microscopic surface.

## 4. Results and Analysis

### 4.1. Effect of Vibration Direction on Grinding Force

**Figure 5:**
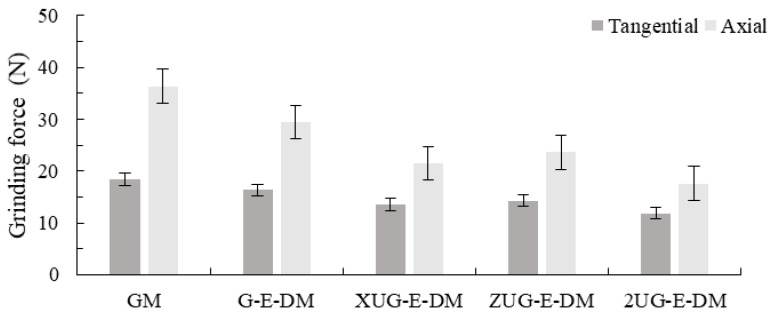
Grinding force in different vibration directions.

The grinding force comparison test results in different vibration directions are shown in Figure 5. It can be seen from the figure that the grinding force of 2UG-E-DM is the smallest, and its tangential force is reduced by 35.79%, 27.63%, 12.76%, and 17.26% compared with the other four processes, while the normal force is reduced by 35.79%, 27.63%, 12.76%, and 17.26%, with the largest decrease in GM and the smallest decrease in XUG-E-DM. After increasing the ultrasonic vibration, this not only increased the discharge frequency, but it also prolonged the actual motion arc length of the grinding, thereby reducing the grinding force [25]. 

In addition, the tangential grinding force and normal grinding force values of ZUG-E-DM are larger than XUG-E-DM. This finding is because although the axial vibration of the workpiece increases the motion arc length in ZUG-E-DM, by increasing the proportion of the plastic shear domain of the material, its elastic deformation and plowing domain increase because the abrasives of the tool are always in contact with the workpiece surface during processing, and the influence of the size effect cause its grinding force to increase instead. The abrasives grind the workpiece surface intermittently in XUG-E-DM, but the actual grinding time and arc length reduce, and its grinding force is slightly larger than that of 2UG-E-DM. Therefore, the two-way combined ultrasonic vibration in 2UG-E-DM combines the advantages of single ultrasonic-assisted machining and better exerts the force reduction effect of ultrasonic vibration, and the tangential force reduction effect is more obvious.

**Figure 6:**
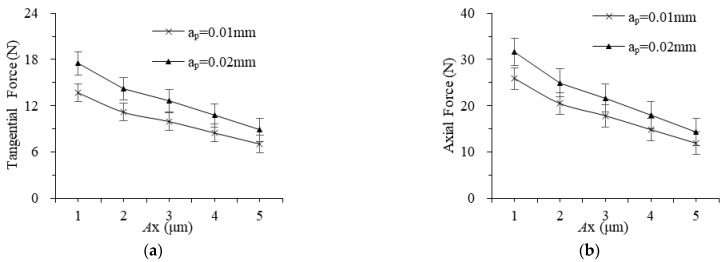
The effect of workpiece vibration amplitude on grinding force: (**a**) tangential and (**b**) axial.

**Figure 7:**
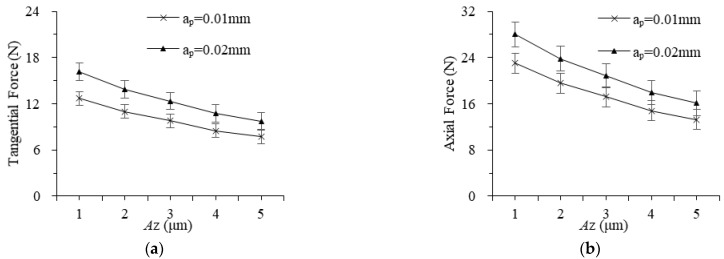
The effect of tool vibration amplitude on grinding force: (**a**) tangential and (**b**) axial.

The grinding forces with different vibration amplitudes of workpieces and tools are shown in Figure 6 and Figure 7. The grinding force decreases almost linearly as the workpiece and tool amplitude increase, and the minimum values of tangential force and normal force are both obtained when the amplitude is 5 μm. When the amplitude of the workpiece is greater, the impact force and indentation depth of the abrasive are greater, more material is removed in the form of “small chips” generated by the ultrasonic impact, and the proportion of plowing and elastoplastic deformation force decreases. When the tool vibration amplitude is larger, the actual motion arc length of an abrasive is longer, which reduces the average grinding force. However, as the amplitude in different directions increases, both the tangential force and the axial force decrease gradually. In addition, the workpiece vibration affects the material removal form of the two-dimensional rotating ultrasonic-assisted grinding, electrolysis, and discharge machining, which plays a key role in whether an abrasive is in the grinding process, and the reduction of grinding force is more significant.

### 4.2. Effect of Vibration Direction on Surface Quality

**Figure 8:**
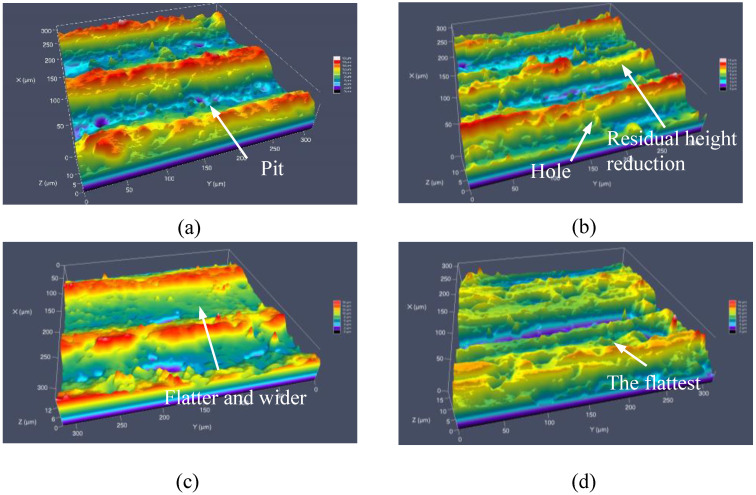
The surface topography of different processes: (**a**) GM; (**b**) G-E-D-M; (**c**) 2UGM; and (**d**) 2UG-E-DM.

The three-dimensional surface morphology after machining is shown in Figure 8. It can be seen from the figure that the surface texture of GM is regular and obvious in Figure 8a. More particles in Figure 8b can be seen at the bottom of the groove of G-E-DM, while the bottom of the pit is rougher than that of GM, and the edge of the groove has more sharp corners which are SiC particles and insoluble substances left by electrolysis discharge. After adding the tool and workpiece vibration of 2UGM in Figure 8c, the grinding domain increases. The residual height is obvious, but the surface quality is improved. This proves that the intermittent impact of an abrasive on the surface of the workpiece under the action of axial ultrasonic vibration leads to an increase in the width of the pit, while the aluminum-based material is squeezed and the SiC particles are dragged and rolled under the action of the tangential ultrasonic vibration, resulting in pits and some defects [26]. The wall surface is smooth and uniform, and the bottom is flat in Figure 8d. The surface morphology after processing has a main texture that is consistent with the relative movement of the tool and the workpiece, and the reciprocating grinding and ironing effects are remarkable. This may be due to the higher efficiency of bidirectional vibration on workpiece material removal, high-frequency softening materials, impact, grinding times, and the surface of the generated pit structure. On the basis of 2UGM, 2UG-E-DM also reduces the height of protrusions and pits due to the electrolytic dissolution of aluminum-based materials.

**Figure 9:**
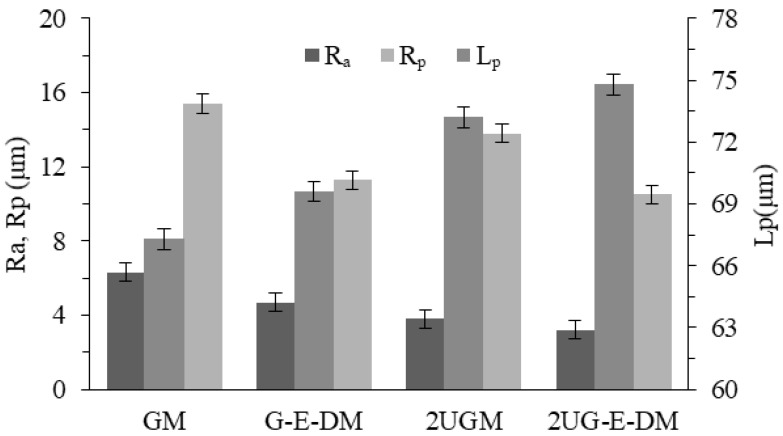
The surface topography parameters of different processes.

The measurement results of surface topography under different processes are shown in Figure 9. The maximum residual height Rp of 2UG-E-DM is about 11.3 μm, which is 4.9 μm, 3.2 μm, and 1.6 μm lower than that of GM, G-E-DM, and 2UGM, respectively. This shows that the ironing and electrolysis of the two-way ultrasound have a significant effect on reducing the residual height. The pit-spacing Lp of GM is about 67.3 μm, and under the action of two-dimensional ultrasound and voltage in 2UG-E-DM, the pit spacing increases to 75.5 μm, which is consistent with the verification results of the surface topography. The Ra of 2UG-E-DM is only 3.2 μm, which is 45.6%, 24.0%, and 9.2% lower than that of GM, G-E-DM, and 2UGM, respectively. The changing trend of the impact analysis on the surface appearance is basically the same, which proves that increasing the two-dimensional ultrasonic vibration and voltage using the broadening effect of axial ultrasonic, the rolling effect of tangential ultrasonic, and the leveling effect of electrolysis discharge effectively reduces the surface roughness. The surface quality is better in the composite process with two-dimensional vibration.

**Figure 10:**
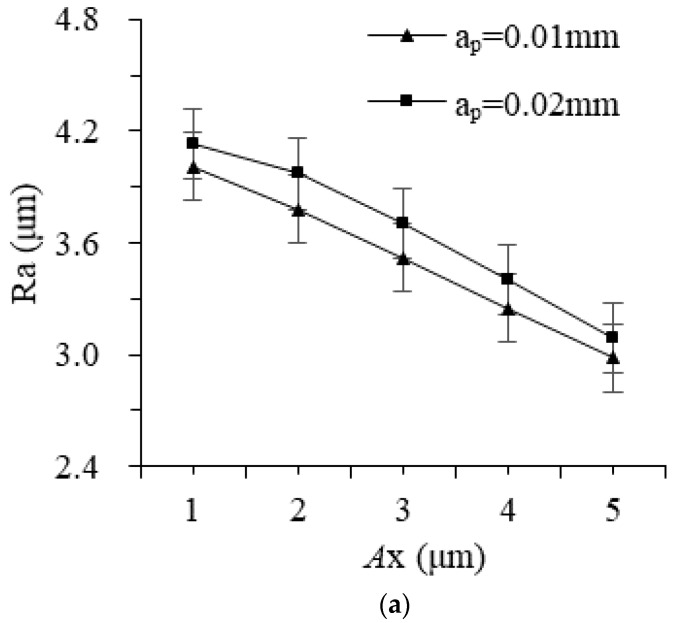

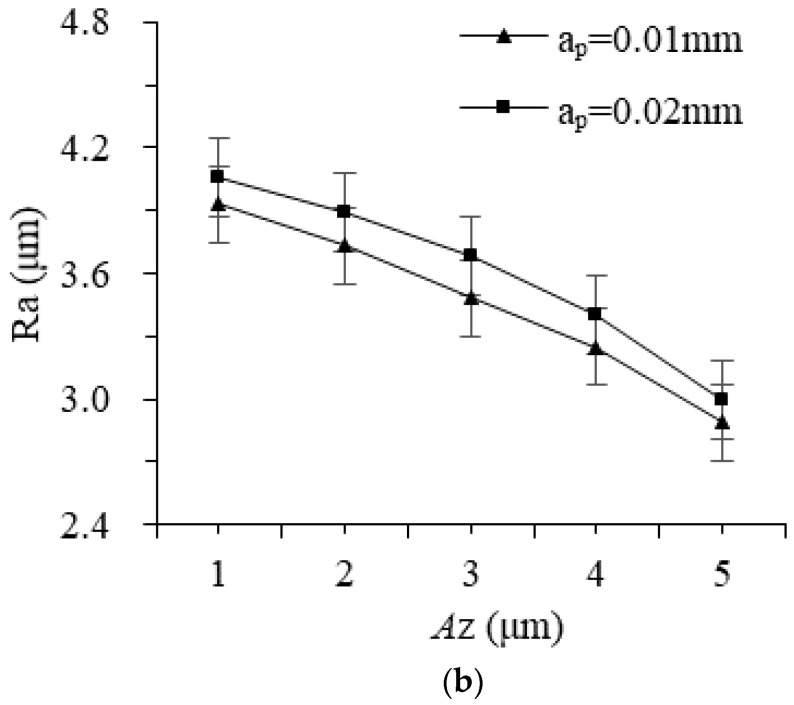
The effect of different amplitudes on surface roughness: (**a**) amplitude of workpiece and (**b**) amplitude of tool.

The change law of surface roughness values under different amplitudes is shown in Figure 10. As the amplitude of the workpieces and tools increases, Ra decreases significantly, and the relationship between the workpiece vibration and the surface roughness is almost linear, while the effect is weaker at a smaller tool amplitude. As the amplitude of the workpiece increases, the dragging displacement of the base particles enhances, the area ratio of the rolling area and the width of the groove increase year-on-year, the residual height between the abrasive particles reduces, and the processed surface improves.

**Figure 11:**
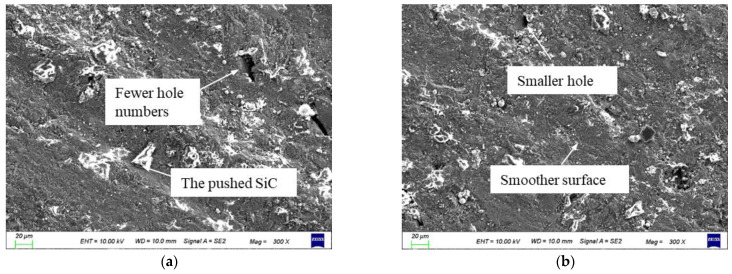
Surface SEM diagrams of different amplitudes: (**a**) No. 2 in Table 3 and (**b**) No. 9 in Table 3.

The SEM images of the machined surface at different amplitudes are shown in Figure 11. Compared with the amplitude of 3 μm, when the amplitude is 5 μm, the protrusion height significantly reduces, the SiC particles that are exposed on the surface are rolled and pushed, the bottom of the pit is rolled and smoothed, the number of holes reduces, and the surface is relatively smaller. Although the electrolytic secondary processing at a large amplitude increases the electrolysis depth and the exposed depth of the residual particles, the two-dimensional rotational ultrasonic rolling of the particles effectively reduces the height difference between the ridges and grooves and reduces the surface roughness value. Ultrasonic action has a more obvious effect on improving the surface quality.

## 5. Simulation of an Abrasive Grinding

Since ultrasonic-assisted grinding of SiCp/Al composite materials reduces brittle fracture and has better surface quality, it is assumed that the material is removed by plastic grinding. ABAQUS 6.14 software was used to analyze the variation law of the single abrasive grinding process [27]. The whole simulation model used a millimeter-unit system. The meshing size was selected for tetrahedral mesh division, and the mesh cell size was set to 0.001 mm. The machining width of the workpiece was 0.1 mm, the machining depth was 0.01 mm, the tool radius was 3mm, and the single abrasive was simplified into a pyramid shape with a height of 0.15 mm. The Johnson–Cook material model was selected to establish the constitutive model, and the parameter values are shown in Table 4 [28]. The Johnson–Cook fracture criterion was selected to simulate the single abrasive grinding process, and the failure parameters are shown in Table 5. We considered the abrasive as a rigid body and designed the simulation analysis model of general grinding (GM), X direction (tangential) ultrasonic-assisted grinding (XUGM), Z direction (axial) ultrasonic-assisted grinding (ZUGM), and two-dimensional ultrasonic-assisted grinding (2UGM). The simulation processing parameters are shown in Table 6.

**Figure 12:**
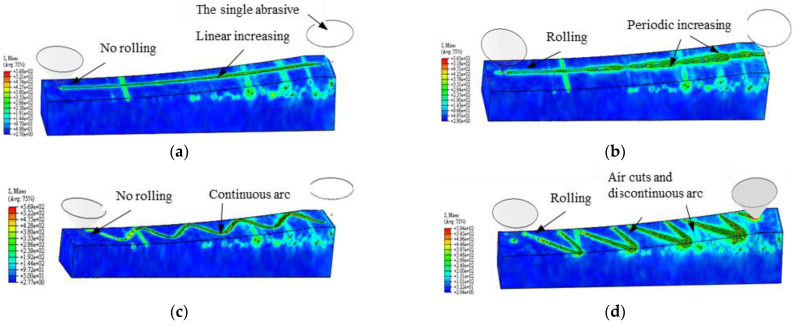
Simulation of an abrasive grinding process: (**a**) GM; (**b**) XUGM; (**c**) ZUGM; and (**d**) 2UGM.

The generative machining simulation of the abrasive under different vibration directions is shown in Figure 12. In Figure 12a, the grinding depth increases linearly from the entry point to the exit point during single abrasive general grinding, and the maximum grinding depth is related to the grinding depth, tool feed speed, and rotation. In the initial stage of contact between the abrasive and the workpiece, the elastic–plastic deformation and plowing area are larger. In Figure 12b, the X direction vibration of the workpiece periodically increases the tangential grinding depth, the tangential maximum grinding depth is generated at the maximum vibration displacement, and there is reciprocating rolling at the entry point of the workpiece vibration. In Figure 12c, the abrasive is always in contact with the workpiece surface, the Z direction ultrasonic vibration increases the grinding arc length and is continuous, the grinding depth increases with the increase in the arc length, and there is no reciprocating grinding of the single abrasive grain at the entry point. This is consistent with Yang’s kinematic trajectory analysis in ultrasonic-vibration-assisted grinding [29]. In Figure 12d, under the X direction vibration of the workpiece, the abrasive periodically contacts the surface of the workpiece, and the arc length is discontinuous. The grinding depth is periodic and intermittent with the grinding arc length under the action of two-way ultrasonic, and the maximum tangential grinding depth is only related to the vibration of the workpiece. The air cutting occurs between two adjacent vibration cycles of the workpiece, and the occurrence of air cutting is related to the vibration frequency of the workpiece with an abrasive in a single effective processing time. In addition, due to the ultrasonic vibration of the workpiece, the abrasive is reciprocally rolled and ground at the entry point in Figure 12b,d.

In the simulation analysis, it can be seen that the vibration of the workpiece produces empty cutting, and the chips are not continuous, but it is precisely because of the existence of empty cutting that the abrasive processing under the two-dimensional ultrasonic vibration has the following grinding characteristics: it periodically impacts the workpiece surface; it increases the tangential grinding depth and reduces the ultrasonic softening effect for the grinding force; it increases the processing coverage area; it abrades the surface of the workpiece; it reduces the average grinding depth and the grinding force; it grinds the surface of the workpiece; it widens the width of the groove marked by the abrasive; it reduces the residual height on the surface; it changes the surface morphology; and it improves the surface quality.

From the above results, it can be seen that the two-dimensional rotational ultrasonic vibration and its vibration direction greatly influence the grinding force and surface quality; additionally, the grinding force is lower and the surface roughness is smaller under the larger amplitude and the auxiliary effect of the two-dimensional vibration. The high strain rate and softening of the material is caused by the single abrasive particle impact grinding process at high speed, high frequency, and large amplitude. However, when the amplitude is small, the softening effect decreases accordingly; the cutting thickness to achieve material removal in the grinding process decreases; the ploughing and elastoplastic deformation increase, which leads to the weakening of the composite machining effect; and the displacement and shedding of SiC particles in the composite material leads to deterioration of the surface quality. Therefore, two-dimensional ultrasonic-assisted machining could be selected to machine difficult-to-machine composite materials and increase the two-dimensional amplitude to reduce the grinding force and improve the surface processing quality.

## 6. Conclusions

In this research, the 2UG-E-DM method was developed for machining hard and brittle conductive materials of MMCs (40% SiCp/Al) in different vibration directions. The experimental research focused on the grinding force and surface quality under different amplitudes of workpieces and tools. According to the tests and simulations, the following detailed conclusions were obtained:

(1) Under the action of ultrasonic vibration, the abrasive hammers, abrades, and grinds the machined surface. Two-dimensional ultrasonic vibration combines the advantages of single ultrasonic-assisted processing, which reduces the force of the ultrasonic vibration. The largest decrease is in GM, and the smallest decrease is in XUG-E-DM, while the tangential force decreases more than the axial.
(2) The machined surface of 2UG-E-DM is formed under the combined action of two-dimensional ultrasonic-assisted grinding, electrolysis, and electrical discharge machining. The reciprocating grinding and smoothing of the surface grooves and ridges by two-dimensional ultrasonic vibration increases the groove width, smooths the surface morphology after grinding, and decreases the height difference of the ridges. The surface roughness is 45.6%, 24.0%, and 9.2% lower than that of GM, G-E-DM, and 2UGM2UGM, respectively.
(3) As the amplitude increases, the axial and tangential forces gradually decrease, and the surface roughness becomes smaller. When the amplitude increases to 5 μm, the decreasing trend of the grinding force weakens. The abrasives reciprocal rolling, widening, and leveling effects of the machined surface grooves are obvious, and the improved processing effect is made more significant as the amplitude of the workpiece increases.

## Figures and Tables

**Table 1:** Physical properties of the composite materials.

Material	Density (g/cm^3^)	Hardness (HRC)	Poisson’s Ratio	Elastic Modulus (Gpa)	Fracture Toughness (Mpa/m^2^)	Thermal Conductivity (W/m∙K)	Elongation
40% SiCp/Al	2.9	32	0.33	163	7.8	183	0.19%

**Table 2:** The comparative test parameters of different processes.

Category	Voltage U (V)	Amplitude of Workpiece A_X_ (μm)	Amplitude of Tool A_Z_ (μm)
**GM**	0	0	0
**G-E-DM**	4	0	0
**XUG-E-DM**	4	4	0
**ZUG-E-DM**	4	0	4
**2UGM**	0	4	4
**2UG-E-DM**	4	4	4

**Table 3:** The processing test parameters of different amplitudes.

No.	X Amplitude of Workpiece A_X_ (μm)	Z Amplitude of Tool A_Z_ (μm)
**1**	1	4
**2**	2	4
**3**	3	4
**4**	4	4
**5**	5	4
**6**	4	1
**7**	4	2
**8**	4	3
**9**	4	5

**Table 4:** Model parameters of the composite materials.

A (MPa)	B (MPa)	n	C	m	Tr (°C)	Tm (°C)
315	520	0.21	0.003	0.843	20	730

**Table 5:** Failure parameters of the composite materials.

D_1_	D_2_	D_3_	D_4_	D_5_
0.118	0.126	−0.374	0.036	0

**Table 6:** Design of the simulation machining parameters.

No.	Category	Feed Speed v_w_ (mm/min)	Spindle Speed n (rpm)	Grinding Depth a_p_ (mm)	X Amplitude AX (μm)	Z Amplitude AZ (μm)
(a)	GM	15	1200	0.01	0	0
(b)	XUGM	15	1200	0.01	0	5
(c)	ZUGM	15	1200	0.01	5	0
(d)	2UGM	15	1200	0.01	5	5

## Data Availability

Research data are available in the paper.

## Nomenclature

2UG-E-DM	Two-dimensional ultrasonic-assisted grinding, electrolysis, and discharge machining
2UGM	Two-dimensional ultrasonic-assisted grinding machining
ECM	Electrolysis machining
EDM	Electro-discharge machining
GM	Grinding machining
G-E-DM	Grinding and electrolysis discharge machining
ZUG-E-DM	Z direction ultrasonic grinding, electrolysis, and discharge machining
XUG-E-DM	X direction ultrasonic grinding, electrolysis, and discharge machining
MMC	Metal matrix composites
Ra	Surface roughness
R_p_	The maximum residual height
L_p_	The length between adjacent bumps
A_Z_	Vibration amplitude of Z direction
f_Z_	Vibration frequency of Z direction
A_X_	Vibration amplitude of X direction
f_X_	Vibration frequency of X direction
n	Spindle speed
Ge(*t*)	Machining gap of electrolysis discharge machining

## References

[1] Natarajan E., Freitas L.I., Santhosh M.S., Markandan K., Al-Talib A.A.M., Hassan C.S. (2023). Experimental and numerical analysis on suitability of S-Glass-Carbon fiber reinforced polymer composites for submarine hull. Def. Technol..

[2] Ansari B., Aligholami M., Rostamzadeh Khosroshahi A. (2022). An experimental and numerical investigation into using hydropower plant on oil transmission lines. Energy Sci. Eng..

[3] Wang X., Xu C., Hu D., Li C., Liu C., Tang Z. (2021). Effect of ultrasonic shot peening on surface integrity and fatigue performance of single-crystal superalloy. J. Mater. Process. Technol..

[4] Sahmani S., Fattahi A.M., Ahmed N.A. (2020). Develop a refined truncated cubic lattice structure for nonlinear large-amplitude vibrations of micro/nano-beams made of nanoporous materials. Eng. Comput..

[5] Zhang X., Yang L., Wang Y., Lin B., Dong Y., Shi C. (2020). Mechanism study on ultrasonic vibration assisted face grinding of Hard and brittle materials. J. Manuf. Process..

[6] Lei X., Xiang D., Peng P., Liu G., Li B., Zhao B., Gao G.G. (2022). Establishment of dynamic grinding force model for ultrasonic-assisted single abrasive high-speed grinding. J. Mater. Process. Technol..

[7] Zhang C., Xu Z., Lu J., Geng T. (2021). An electrochemical discharge drilling method utilising a compound flow field of different fluids. J. Mater. Process. Technol..

[8] He B., Li H., Ma X., Li J., Fan S.S. (2021). Plane Machining by Inner-Jet Electrochemical Milling of TiB_2_/7050 Aluminum Matrix Composite. Appl. Sci..

[9] Elhami S., Razfar M.R. (2020). Application of nano electrolyte in the electrochemical discharge machining process. Precis. Eng..

[10] Sui H., Zhang X., Zhang D. (2021). Surface modeling and analysis of high-speed ultrasonic vibration cutting. Mach. Sci. Technol..

[11] Li Z., Ming Z., Xiong H., Zhou J. (2019). 3D surface roughness evaluation of surface topography in ultrasonic vibration assisted end grinding of SiCp/Al composites. Int. J. Nanomanuf..

[12] Shen X.H., Shi Y.L., Zhang J.H., Zhang Q.J., Tao G.C., Bai L.J.J. (2020). Effect of process parameters on micro-textured surface generation in feed direction vibration assisted milling. Int. J. Mech. Sci..

[13] Zhou Z., Zheng Q., Ding C., Yan J., Peng G., Piao Z. (2021). Research on the promotion mechanism of surface burnishing process by two-dimensional ultrasonic vibration. J. Mater. Res. Technol..

[14] Zhang Y., Li C., Ji H., Yang X., Yang M., Jia D., Wang J. (2017). Analysis of grinding mechanics and improved predictive force model based on material-removal and plastic-stacking mechanisms. Int. J. Mach. Tools Manuf..

[15] Shi H., Yuan S., Zhang C., Chen B., Li Q., Li Z., Qian J. (2019). A cutting force prediction model for rotary ultrasonic side grinding of CFRP composites considering coexistence of brittleness and ductility. Int. J. Adv. Manuf. Technol..

[16] Cong W., Pei Z., Sun X., Zhang C. (2014). Rotary ultrasonic machining of CFRP: A mechanistic predictive model for cutting force. Ultrasonics.

[17] Li Z., Yuan S., Song H., Batako A.D. (2018). A cutting force model based on kinematics analysis for C/SiC in rotary ultrasonic face machining. Int. J. Adv. Manuf. Technol..

[18] Zha H., Feng P., Zhang J., Yu D., Wu Z. (2018). Material removal mechanism in rotary ultrasonic machining of high-volume fraction SiCp/Al composites. Int. J. Adv. Manuf. Technol..

[19] Wang H., Zhang D., Li Y., Cong W. (2020). The effects of elliptical ultrasonic vibration in surface machining of CFRP composites using rotary ultrasonic machining. Int. J. Adv. Manuf. Technol..

[20] Gao T., Zhang X., Li C., Zhang Y., Yang M., Jia D., Zhu L.L. (2020). Surface morphology evaluation of multi-angle 2D ultrasonic vibration integrated with nanofluid minimum quantity lubrication grinding. J. Manuf. Process..

[21] Liu J., Jiang X., Han X., Gao Z., Zhang D. (2018). Effects of rotary ultrasonic elliptical machining for side milling on the surface integrity of Ti-6Al-4V. Int. J. Adv. Manuf. Technol..

[22] Liu J., Yue T., Guo Z. (2013). Grinding-aided electrochemical discharge machining of particulate reinforced metal matrix composites. Int. J. Adv. Manuf. Technol..

[23] Singh T., Dvivedi A. (2018). On performance evaluation of textured tools during micro-channeling with ECDM. J. Manuf. Process..

[24] Li J., Chen W., Zhu Y. (2022). Study on Generating Machining Performance of Two-Dimensional Ultrasonic Vibration-Composited Electrolysis/Electro-Discharge Technology for MMCs. Materials.

[25] Zhao B., Chang B., Wang X., Bie W. (2019). System design and experimental research on ultrasonic assisted elliptical vibration grinding of Nano-ZrO_2_ ceramics. Ceram. Int..

[26] Natarajan E., Markandan K., Sekar S.M., Varadaraju K., Nesappan S., Albert Selvaraj A.D., Franz G. (2022). Drilling-Induced Damages in Hybrid Carbon and Glass Fiber-Reinforced Composite Laminate and Optimized Drilling Parameters. J. Compos. Sci..

[27] Xiang D., Shi Z., Feng H., Wu B., Zhang Z., Chen Y., Zhao B. (2019). Finite element analysis of ultrasonic assisted milling of SiCp/Al composites. Int. J. Adv. Manuf. Technol..

[28] Wang J., Yin Y., Luo C. (2018). Johnson–Holmquist-II (JH-2) constitutive model for rock materials: Parameter determination and application in tunnel smooth blasting. Appl. Sci..

[29] Yang Z., Zhu L., Ni C., Ning J. (2019). Investigation of surface topography formation mechanism based on abrasive-workpiece contact rate model in tangential ultrasonic vibration-assisted CBN grinding of ZrO_2_ ceramics. Int. J. Mech. Sci..

